# Molecular and Mechanobiological Pathways Related to the Physiopathology of FPLD2

**DOI:** 10.3390/cells9091947

**Published:** 2020-08-23

**Authors:** Alice-Anaïs Varlet, Emmanuèle Helfer, Catherine Badens

**Affiliations:** 1 Marseille Medical Genetics (MMG), INSERM, Aix Marseille University, 13005 Marseille, France; alice-anais.varlet@univ-amu.fr; 2 Centre Interdisciplinaire de Nanoscience de Marseille (CINAM), CNRS, Aix Marseille University, 13009 Marseille, France

**Keywords:** lamin A/C, FPLD2, laminopathies, signaling pathway, adipocytic cells, endothelial cells, cell mechanical properties

## Abstract

Laminopathies are rare and heterogeneous diseases affecting one to almost all tissues, as in Progeria, and sharing certain features such as metabolic disorders and a predisposition to atherosclerotic cardiovascular diseases. These two features are the main characteristics of the adipose tissue-specific laminopathy called familial partial lipodystrophy type 2 (FPLD2). The only gene that is involved in FPLD2 physiopathology is the *LMNA* gene, with at least 20 mutations that are considered pathogenic. *LMNA* encodes the type V intermediate filament lamin A/C, which is incorporated into the lamina meshwork lining the inner membrane of the nuclear envelope. Lamin A/C is involved in the regulation of cellular mechanical properties through the control of nuclear rigidity and deformability, gene modulation and chromatin organization. While recent studies have described new potential signaling pathways dependent on lamin A/C and associated with FPLD2 physiopathology, the whole picture of how the syndrome develops remains unknown. In this review, we summarize the signaling pathways involving lamin A/C that are associated with the progression of FPLD2. We also explore the links between alterations of the cellular mechanical properties and FPLD2 physiopathology. Finally, we introduce potential tools based on the exploration of cellular mechanical properties that could be redirected for FPLD2 diagnosis.

## 1. Introduction

In eukaryotic cells, the lamina, localized underneath the inner membrane of the nuclear envelope (NE), corresponds to a meshwork of type V intermediate filaments, called lamin proteins, and connects to lamin-associated proteins, such as the linker of nucleoskeleton and cytoskeleton (LINC) complex constituents and the nuclear pore complexes [1]. Lamins are divided into two groups, according to their sequence homology and biochemical properties, namely, the A-type lamins encoded by the *LMNA* gene and the B-type lamins encoded by the *LMNB1* and *LMNB2* genes [2]. 

A-type lamins, corresponding to lamin A and C proteins (referred to hereafter as lamin A/C), are related to three main functions. First, by controlling the lamina meshwork organization, the lamin A/C protein maintains the nuclear mechanical stability, shape and rigidity [3,4]. Second, lamin A/C associates with heterochromatin through its interaction with lamin-associated domains, which are typically repressive regions in the genome [5,6,7,8]. Through this association, lamin A/C influences the chromatin structure and organization, as well as gene silencing. Third, lamin A/C is also present in the nucleoplasm, where it interacts with and regulates transcription factors. This consequently relates lamin A/C to key signaling pathways, including those regulating the cell cycle and DNA repair [9,10]. Through these three primary functions, lamin A/C contributes to sensing and responding to mechanical cues from the cytoplasm—two processes named mechanosensing and mechanotransduction, respectively. As most studies describing lamin A/C functions have not discriminated between the role of lamin A and lamin C, the precise role of each protein is still not well-understood. 

There is a significant difference in the post translational steps for lamin A and lamin C processing: Whereas lamin C is produced in its definite form, lamin A undergoes key post-translational modifications required for its proper incorporation into the lamina meshwork in its final form. Indeed, as a precursor called prelamin A, the protein experiences processing, including farnesylation, methylation and cleavage by the metalloprotease ZMPSTE24 [11]. Interfering with the cleavage steps leads to the accumulation of farnesylated prelamin A, which remains anchored to the nuclear membrane, while mature lamin A does not. The accumulation of prelamin A is toxic for cells and is associated with several cellular phenotypes, such as DNA repair defects, oxidative stress and premature senescence, corresponding to the progressive decline of cellular functions ending in cell cycle arrest [12,13,14,15]. 

Numerous mutations in either of the genes encoding the enzymes involved in this process or in *LMNA* itself could be the cause of the pathologic inhibition of prelamin A maturation [16,17,18]. Other mutations of *LMNA* alter lamin A/C’s function in a way which is not clearly understood. Diseases associated with *LMNA* mutations or lamin A processing alterations are gathered in a family of pathologies and called laminopathies. More than 15 pathologies have been included in laminopathies, which can be multi-systemic or tissue specific. The multi-systemic laminopathy Progeria, which is the most severe type, is caused by the accumulation of a prelamin A mutant, called progerin, which results from a deletion of 50 amino acids near the C-terminus of lamin A [19,20]. Progeria patients present a distinctive appearance, characteristics of premature aging such as alopecia and thin skin with visible veins, a loss of subcutaneous fat and muscle mass, insulin resistance and cardiovascular symptoms related to atherosclerosis. Tissue-specific laminopathies usually affect a single tissue: muscular tissue, as is the case for Emery–Dreifuss muscular dystrophy [21]; cardiac tissue, as is the case for dilated cardiomyopathy type 1A (DCM-1A) [22]; or adipose tissue, as is the case for type 2 familial partial lipodystrophy (FPLD2). 

At the clinical level, patients with multi-systemic or tissue-specific laminopathies present a wide range of clinical signs that can be shared by several laminopathies or be specific to one type of laminopathy [11]. For example, the increased risk for atherosclerosis observed in Progeria is a feature also observed in Mandibuloacral Dysplasia, another multi-systemic laminopathy, and in FPLD2 [23]. At the cellular level, several multi-systemic and tissue-specific laminopathies are characterized by a decrease in the cell proliferation rate, premature senescence, misshapen nuclei and chromatin remodeling in one up to almost all cell types [24]. Importantly, laminopathies constitute a strong model for exploring the mechanisms involved in the senescence process, as most clinical and cellular features characteristic of the pathologies are shared with normal aging. 

FPLD2 or Dunnigan syndrome is a tissue-specific laminopathy affecting the adipose tissue and characterized by a loss of subcutaneous adipose tissue in the trunk, buttocks and limbs; fat accumulation in the neck, face, axillary and pelvic regions; apparent muscular hypertrophy; and metabolic dysfunctions (MDs), such as insulin resistance, diabetes mellitus, dyslipidemia and liver steatosis. MDs in FPLD2 are associated with a high incidence of cardiovascular diseases, such as atherosclerosis and coronary disease [25]. FPLD2 results from at least 20 *LMNA* missense mutations; however, the recurrent p.R482W/Q substitutions are responsible for 80% of the cases. As the main MD features of FPLD2 are shared by multi-systemic laminopathies, understanding the molecular mechanisms driving FPLD2 physiopathology represents a major opportunity for exploring potential therapeutic avenues in the field of laminopathies.

In this review, we will present the molecular mechanisms that have been associated with the physiopathology of MDs and cardiovascular diseases in the context of FPLD2. More precisely, we will focus on the effects of FPLD2-associated *LMNA* mutations on adipocyte and endothelial cell differentiation and we will discuss some questions that remain unanswered. We will also report on the potential functional link, remaining to be explored, between the mechanical properties of the lamin A/C meshwork and the effect of FPLD2-associated *LMNA* mutations on adipose and vascular tissue premature aging. Finally, we will introduce techniques for mapping cellular mechanical properties that could be exploited as diagnostic tools to identify patients affected by FPLD2 or severe MDs with *LMNA* mutations.

## 2. Signaling Pathways Affected by FPLD2-Associated *LMNA* Mutations That Contribute to the Onset of MD and Cardiovascular Disease

### 2.1. Abnormal Sequestration of the Transcription Factors SREBP1 and Sp1, Caused by FPLD2-Associated LMNA Mutations, Inhibits Adipocytic Differentiation

Sterol response element binding protein 1 (SREBP1) belongs to the SREPB family of membrane-bound transcription factors, which are significant regulators of cholesterol and fatty acid homeostasis [26]. SREBP1 is a well-known adipocytic factor whose activation is crucial for the initiation of mesenchymal stem cell (MSC) differentiation into adipocytes [27]. 

In 2002, this transcription factor was identified as a novel interactor of lamin A using a yeast two-hybrid interaction screen. Through glutathione S-transferase pull-down, it was shown that the binding of lamin A to SREBP1 was noticeably reduced by the FPLD2-associated mutations G465D, R482W and K486N [28]. For the first time, this study suggested that MDs observed in FLPD2 may be caused, at least in part, by the reduced binding of the adipocytic differentiation factor SREBP1 to lamin A. Later, an interaction of SREBP1 with prelamin A, but not with mature lamin A/C, was confirmed in patient fibroblasts and control fibroblasts forced to accumulate prelamin A by treatment with an inhibitor of farnesylation [29]. Additionally, the authors found that prelamin A specifically accumulated in lipodystrophic cells and co-localized at the NE with SREPB1. To understand the effect of prelamin A accumulation in adipose tissue, the authors inhibited lamin A/C maturation in 3T3-L1 pre-adipocytes and showed that prelamin A sequestered SREBP1 at the nuclear circumference. The results suggested that an abnormal amount of prelamin A in lipodystrophic cells could decrease the pool of active SREBP1 by sequestration and impair adipocytic differentiation. 

The sequestration model was supported by Duband-Goulet et al., who showed that, in HeLa cells, overexpressed wild type (WT) and lipodystrophy-associated mutants of lamin A/C (R482W) bound to SREBP1 and favored its localization at the nuclear periphery [30]. 

The transcription factor sequestration model proposed for SREBP1 might be a general model of the pathway to MDs when *LMNA* mutations lead to prelamin A accumulation. Indeed, it was described that such accumulation artificially leads to the sequestration and inhibition of the transcription factor Sp1, known to regulate the extracellular matrix (ECM) and lipid metabolism genes [31]. Sp1 sequestration resulted in altered ECM-related gene expression and interfered with human MSC adipocytic differentiation. 

An outstanding study completed the sequestration model proposed, using complementary approaches to show that lamin A/C preferentially interacted with SREBP1 bound to DNA rather than free SREBP1 [32]. Therefore, the study suggested that A-type lamins may associate with and displace DNA-bound SREBP1 to a transcriptionally inactive nuclear compartment, such as the NE. The authors confirmed that the overexpression of WT lamin A/C or the R482W mutant reduced the transcriptional activity of SREBP1 in HeLa cells, as suggested by Duband-Goulet et al. [30]. 

However, both in human fibroblasts and adipose progenitor cells, the same study showed that the expression of the R482W mutant specifically elicited the up-regulation of numerous SREBP1 target genes, in correlation with a marked decrease in the number of lamin A/C–SREBP1 in situ interactions revealed by proximity ligation assay (PLA) experiments [32]. The fact that a specific impact of the R482W mutant on SREBP1 was observed in only two of the three cellular models used in the study suggests that cell-type specificity could contribute to the regulation of SREBP1’s function. 

In summary, SREBP1, as well as Sp1, seem to be significant partners of lamin A/C in the process of adipocytic differentiation. However, the precise molecular mechanism by which A-type lamins modulate these transcription factor activities has not yet been fully elucidated. Additionally, it remains important to confirm the model of transcription factor sequestration as a common pathway to FPLD2 physiopathology. In addition to a potential effect on SREPB1 and Sp1 transcription factors, researchers have proposed that *LMNA* mutations associated with FPLD2 interfere with key signaling pathways involved in adipocytic differentiation. We will describe this in more detail in the next section. 

### 2.2. Altered Adipocytic Differentiation Is Triggered by a Misregulation of the Notch and Wnt/β-Catenin Pathways 

Among the several signaling pathways identified in the process of adipocytic differentiation, the Wnt/β-catenin and Notch pathways are the best-known. These two axes play key roles not only in regulating adipocyte progenitor cell proliferation and differentiation in vitro, but also in modulating adipose development and functions, beige adipocyte formation and the whole body energy metabolism in vivo [33,34]. Both signaling pathways induce osteoblastogenesis (a process leading to the expression of osteogenic markers in adipocytes) and block the induction of peroxisome proliferator-activated receptor-γ (PPAR-γ), which is a prime inducer of adipocytic differentiation.

**Figure 1:**
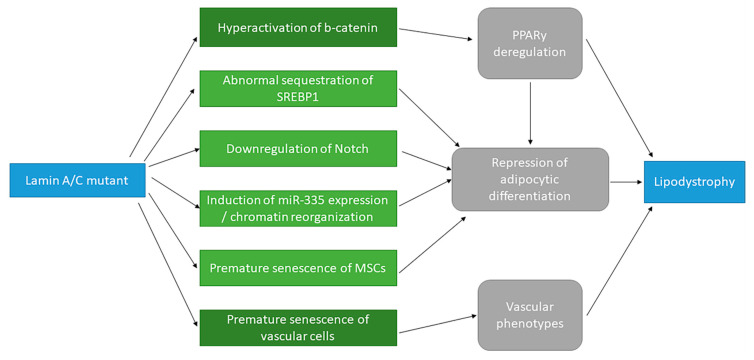
Summary scheme of the different signaling pathways affected by *LMNA* mutations associated with the physiopathology of familial partial lipodystrophy type 2 (FPLD2).

Recently, the role of the R482L mutant in modulating Notch signaling activity during the adipocytic differentiation of MSCs was investigated [35]. MSCs were transduced with lentiviruses bearing *LMNA* WT or *LMNA* p.R482L and with the Notch-intracellular domain to activate Notch signaling and induce adipocytic differentiation. In this context, it was highlighted, using qPCR and luciferase-reporter techniques, that when Notch is activated, the R482L mutant, conversely to the WT lamin A/C, contributed to the down-regulation of Notch activation in undifferentiated and differentiated cells, and decreased the adipocytic differentiation. Therefore, the results suggest that lamin A/C influences adipocytic differentiation through Notch signaling and that *LMNA* mutations associated with FPLD2 could cause alterations of this process (Figure 1). However, it is important to note that all experiments in this study were performed in a non-physiological context: The overexpression of the Notch-intracellular domain. Consequently, it is essential to confirm the authors’ observations in vivo.

In 2006, Boguslavsky et al. were the first to investigate the potential role of lamin A/C in adipocytic differentiation, depending on PPAR-γ expression [36]. By analyzing the differentiation of 3T3-L1 preadipocytes, the authors showed that the overexpression of both WT lamin A/C and R482Q/W mutants inhibited lipid accumulation, triglyceride synthesis and the expression of adipocytic markers. This was associated with the inhibition of PPAR-γ, suggesting that A-type lamins act as inhibitors of adipocytic differentiation by affecting PPAR-γ and, potentially, the Wnt/β-catenin pathway. This hypothesis was tested by assessing the effect of lamin A/C overexpression on MSC differentiation [37]. Adipocytic differentiation and the expression of adipocytic factors were significantly lower in transfected cells, confirming the results obtained by Boguslavsky et al. The nuclear β-catenin expression level was significantly higher and the protein was more active in MSCs overexpressing lamin A/C, indicating that these cells presented a lower adipocytic differentiation potential. 

How lamin A/C influences β-catenin nuclear signalization was later investigated by Markiewicz’s group, who suggested that adipocytic differentiation required a fine-tuning of the nuclear emerin, lamin A/C and β-catenin expression levels [38]. In this model, the upregulation of emerin could contribute to the efficient re-distribution of β-catenin from the nucleus to the cytoplasm. These changes could facilitate the proteasomal degradation of β-catenin and, consequently, the activation of PPAR-γ [39]. In addition to β-catenin proteasomal degradation, the downregulation of both β-catenin and emerin, accompanied by the increased expression of lamin A/C [40], could also result in the early onset of PPAR-γ activation and, thus, adipocytic differentiation. 

Overall, these studies suggested that lamin A/C plays a critical role in adipocytic differentiation, potentially through the activation of Notch and β-catenin, which may depend on the lamin A/C and emerin expression levels (Figure 1). However, the molecular mechanism controlling the expression level of these proteins during adipocytic differentiation is still not well-understood. One of the hypotheses states that adipocytic differentiation depends on ECM stiffening, which directly influences the lamin A/C expression level [41]. This model will be explored in more detail later in this review (Section 3.1). Among the molecular processes involved in adipocytic differentiation that might be affected by *LMNA* mutations, the regulation of miRNAs seems to provide new leads to explore and will be described hereafter.

### 2.3. Overexpression of miR-335 Driven by FPLD2-Associated LMNA Mutations Interferes with Adipocytic Differentiation

miRNAs are short, non-coding RNAs that commonly down-regulate targeted mRNAs through their degradation or translational silencing after binding to their 3′ UTR. Numerous studies have described the relationship between certain miRNAs and several main features of MD, such as insulin resistance, obesity, diabetes, lipid metabolism, hypertension, hyperuricemia, and stress [42]. Among these miRNAs, miR-335 represents an important player as it inhibits MSC differentiation into adipocytes and osteocytes, and is involved in mesendodermal and chondrogenic induction [43,44,45]. 

Recently, a link between miR-335 and the physiopathology of FPLD2-associated *LMNA* mutations was shown. A substantial increase in the miRNA expression level was described in FPDL2-fibroblasts from five different patients carrying the *LMNA* p.R482W mutation and adipose stem cells overexpressing the lamin A/C R482W mutant [46]. This suggested that high levels of miR-335 were implicated in FPLD2 physiopathology, which was supported by the inhibition of adipocytic differentiation when the miR-335 expression level was decreased [47]. 

ChIP-qPCR experiments allowed an exploration of the molecular mechanism underlying the relationship between the miR-335 expression level and FPLD2 physiopathology [46]. These experiments highlighted that the binding of lamin A/C to the miR-335 locus after the induction of adipocytic differentiation coincided with enhanced H3K27 trimethylation and transcriptional repression of the miRNA. Comparatively, in cells expressing the R482W mutant, the opposite effects were observed: The loss of lamin A/C-miR-335 binding after the induction of the differentiation of cells expressing the mutant enabled H3K27 acetylation on several enhancer sites. This favored a looping of these enhancers onto the miR-335 promoter, as shown by fluorescence in situ hybridization (FISH) experiments, enabling a persistence of miR-335 transcription. Consequently, this impressive work suggests that the overexpression of miR-335 prevents adipocytic differentiation, which is consistent with the lipoatrophy phenotype associated with FPLD2 (Figure 1). 

Interestingly, FISH experiments in this work suggest that the expression of the R482W mutant of lamin A/C induces a reorganization of the chromatin that favors miR-335 expression. This supposes, in turn, that the mutation affects the role of lamin A/C in chromatin organization [5,48,49,50,51,52,53], a point which is supported by the elegant work conducted by Paulsen et al., who mapped lamin-associated domains (LADs) by ChIP-seq and modeled the 3D organization of the chromatin in fibroblasts from patients carrying the *LMNA* p.R482W mutation [54]. The study showed that specific LADs, named “gain” LADs by the authors, only identified in patient fibroblasts and not in control ones, are localized more centrally in the nucleus than all other LADs. In contrast, LADs unique to control fibroblasts (“lost” in patient cells) are found at the nuclear periphery, to the same extent as all LADs in these cells. By analyzing the gene expression levels, the authors pointed out that gain LADs are downregulated, while lost LADs are upregulated, thus providing functional explanations for the 3D chromatin reorganization observed in FPLD2 cells. Therefore, this impressive work suggests that a significant number of genes could be reprogrammed due to chromatin organization changes in response to the lamin A/C R482W mutant (Figure 1). 

A critical next step will be to address the contribution of 3D chromatin reorganization in adipocytic differentiation in vitro and in vivo and more importantly, in the disease phenotype. Research can determine which other loci and miRNAs could be controlled by lamin A/C and if they are involved in the physiopathology of FPLD2. One of the main features associated with FPLD2 and other laminopathies’ physiopathology is tissues’ premature aging as a consequence of prelamin A or progerin accumulation. In the next section, we will focus on the premature senescence of MSCs and vascular tissues, representing the most important cellular types involved in the maintenance and integrity of adipose tissue, whose exhaustion has been linked to the progression of MD.

### 2.4. Accumulation of Prelamin A: A Path to the Premature Senescence of MSCs and Vascular Cells 

The accumulation of prelamin A is a hallmark of severe laminopathies and is associated with cellular premature senescence [55]. This accumulation results from a blocking of the post-translational maturation process that prelamin A should undergo to produce lamin A/C. The cause for alteration is clear in Progeria as the mutant prelamin A, called progerin, lacks the site for ZMPSTE24 cleavage and consequently retains its farnesylated tail, instead of being shortened. For the other missense mutations involved in laminopathies, the mechanism is not well-understood; however, it is assumed that the amino acid change lowers the efficiency of the maturation process and produces a certain amount of unprocessed prelamin A. 

In all cases, this farnesylated prelamin A remains anchored at the NE instead of being released in the lamina meshwork or nucleoplasm, leading to a disorganized NE and an alteration of the lamin A/C physiological functions. Extensive studies on Progeria have shown that the accumulation of prelamin A is markedly associated with regenerative dysfunctions leading to the premature senescence of stem cells [56]. In numerous other conditions, such as FPLD2 and severe MD, patient cells have been described as accumulating prelamin A [29]. As MSCs are the precursors of adipocytes, multiple studies have looked at the build-up effect of prelamin A accumulation on senescence in this cell type. In several studies, the accumulation of prelamin A, artificially induced in MSCs, led to multiple phenotypes associated with cellular senescence, including p16 expression, β-galactosidase activation, cell cycle arrest, replication inhibition, increased lipolysis, mitochondrial dysfunction, endoplasmic reticulum stress, telomere shortening, DNA-damage signaling activation and reactive oxygen species (ROS) elevation [14,57,58]. 

It was underlined that the combination of prelamin A accumulation and stress conditions (i.e., increased lipolysis, mitochondrial dysfunction and endoplasmic reticulum stress) enhanced the senescent phenotype of MSCs, by interfering with autophagy and dysregulating the activity of the octamer binding protein Oct-1 [14]. In WT MSCs, as expected, starvation induced an increase of the autophagic marker LC3 II. Such an increase was higher after treatment with bafilomycin, which is an inhibitor of lysosomal degradation. MSCs expressing prelamin A also presented an increase of LC3 II after starvation. However, bafylomincin treatment did not induce a higher increase of the autophagic marker expression level, suggesting that the autophagic flux is altered in response to prelamin A accumulation. Additionally, the overexpression of Oct-1, coupled with luciferase assays, showed that Oct-1′s activity is downregulated under starvation and prelamin A accumulation. 

Recently, a study described that progerin overexpression or ZMPSTE24 depletion provoked the GATA Binding Protein 4 (GATA4)-dependent expression of chemoattractant protein-1 (MCP-1), leading to the premature senescence of MSCs [59]. This molecular process was associated with an impaired degradation of GATA4 by autophagy in progerin-expressing MSCs. 

Finally, an investigation of the senescent process during the in vitro expansion of stem cells obtained from amniotic fluids revealed other clues. The expression of pluripotency genes and the proliferation rate were shown to be negatively correlated with the content of ROS, DNA damage signs and the onset of premature senescence markers, including the accumulation of prelamin A [58]. The immunoprecipitation of NADPH oxidase isoform 4 (Nox4)—a specific source of ROS—from extracted nuclei, combined with mass spectrometry, hinted at the interaction of Nox4 with lamin A/C. In exploring the molecular relevance of this interaction, the authors emphasized that prelamin A interacted with and sequestered Nox4 in promyelocytic leukemia nuclear bodies, where both proteins appeared to be sumoylated and targeted to proteasomal degradation.

The abnormal senescence of endothelial and smooth muscle cells was also characterized as a typical feature of laminopathies and was largely associated with symptomatic atherosclerosis and cardiovascular diseases. Several studies described the onset of senescent phenotypes by inducing prelamin A accumulation in endothelial or vascular smooth muscle cells using drug treatments or siRNA targeting ZMPEST24 [13,60,61,62]. To the best of our knowledge, only two studies have investigated the effect of *LMNA* mutations associated with FPLD2 physiopathology on senescent phenotypes of endothelial and vascular smooth muscle cells. 

First, the effects of the R482W mutant in comparison to the WT prelamin A overexpression in human coronary artery endothelial cells was analyzed. Exogenous WT prelamin A was correctly processed and localized, whereas the lamin A/C R482W mutant accumulated abnormally at the NE [63]. Additionally, only the R482W mutant induced endothelial dysfunctions, such as decreased NO production, the promotion of the endothelial adhesion of peripheral blood mononuclear cells, the induction of oxidative stress, DNA damage and inflammation. 

A second study demonstrated that the R482W mutant induced endothelial differentiation defects in a developmental model of FPLD2 [64]. This model highlighted that the expression of the lamin A/C mutant led to a repositioning of the mesodermal regulator T/Brachyury locus toward the nuclear center, suggesting an enhanced activation propensity of the locus. When addressing this issue, the authors reported phenotypic and transcriptional alterations in the mesodermal and endothelial differentiation of induced pluripotent stem cells generated from a patient with FPLD2 associated with the *LMNA* p.R482W mutation. Importantly, correction of the *LMNA* mutation rescued p.R482W-associated phenotypes and gene expression and transcriptomic analysis linked endothelial differentiation defects to decreased polycomb-mediated repression of the T/Brachyury locus and the over-activation of targeted genes, such as vascular genes. Therefore, by connecting a lipodystrophic *LMNA* mutation to a disruption of early mesodermal gene expression and defective endothelial differentiation, this study suggested that lamin A/C rewires the fate of several lineages, resulting, when mutated, in multi-tissue pathogenic phenotypes.

Altogether, these studies suggest that prelamin A accumulation drives the premature senescence of stem cells, which could constitute the primary cause of MD and cardiovascular disease development in FPLD2 and in laminopathies in general (Figure 1). As stated before, prelamin A accumulation is not described for all *LMNA* mutations, while cell senescence is constantly observed, suggesting that other pathways to senescence might be implicated. Therefore, other molecular pathways affecting adipocytic and vascular differentiation and activity should be investigated. Among these pathways, altered autophagic degradation appears to be a common molecular mechanism for laminopathies that should be scrutinized in more detail [14,65,66,67,68,69]. This process, which involves the mTOR pathway, will be described in detail hereafter. We will also introduce two molecular mechanisms that could be involved, namely, the retinoblastoma (Rb)-E2F axis and the transmembrane protein triggering receptor expressed on myeloid cells 2 (TREM2).

### 2.5. Potential Targets to Explore in the Physiopathology of MD in the Context of FPLD2 Associated with LMNA Mutations

The mTOR signaling pathway displays a critical role in the regulation of adipocytic differentiation, lipid metabolism, thermogenesis and adipokine synthesis/secretion [70]. Notably, mTOR inhibition leading to the activation of autophagy contributes to white adipose tissue formation. Consistently, a partial knockdown of mTOR increases adipocytic differentiation, although complete inhibition of its activity, as well as the inactivation of p70S6 kinase 1 (pS6K1)—a major target of the mTOR axis—impairs adipocytic differentiation. Several genes, including the one encoding PPAR-γ previously introduced as a master regulator of adipocytic differentiation, are upregulated by mTOR inhibition.

Lamin A/C contributes to the regulation of autophagic flux through the modulation of mTOR activity. Indeed, using a KO mouse model for *LMNA*, Kennedy’s group described that the absence of lamin A/C activated the mTOR signaling axis associated with laminopathy phenotypes, namely, an impaired cardiac function and skeletal muscle dystrophy [71]. Importantly, treatment with rapamycin, which is a specific mTOR-inhibitor, contributed to an improvement of *LMNA*^–/–^ mice phenotypes and an elevation of survival, showing that mTOR activation might mediate phenotypes involved in laminopathy diseases. This hypothesis was supported by multiple studies showing that rapamycin or analogue treatments improved the phenotypes of cell and mouse models of *LMNA* mutation-associated laminopathies [72,73,74,75,76,77,78,79,80]. 

Rapamycin treatment was also related to an amelioration of MD associated with *LMNA* mutations in vitro and in vivo. Indeed, in the context of *LMNA*^–/–^ mice, it was demonstrated that the inhibition of mTOR by rapamycin treatment increased the body weight and fat content, which are two phenotypes linked to MD [73]. To the best of our knowledge, this study was the first and only one to investigate the relationship between mTOR signaling and MD in relation to *LMNA* mutations. Therefore, it is important to understand how lamin A/C regulates mTOR activation and what the effects of *LMNA* mutations associated with FPLD2 physiopathology are on this pathway. 

Additionally, the Rb-E2F axis is a key sensor of metabolism at cellular and organismal levels. Indeed, Rb is considered the pocket protein that represses the transcriptional activity of E2F—a positive regulator of adipocytic differentiation through the control of PPAR-γ [81,82]. Twenty years ago, a molecular mechanism involving the lamin A/C partner lamina-associated polypeptide 2α (LAP2α) in the regulation of Rb in preadipocytes was described [83]. The study demonstrated that, in vivo, LAP2α associated with and impaired promoter sequences in endogenous E2F/Rb-dependent target genes. In addition, the expression of LAP2α in proliferating preadipocytes caused the accumulation of hypophosphorylated Rb, which is indicative of non-cycling cells, and initiated partial differentiation. As LAP2α is a critical partner of lamin A/C, it is relevant to think that the intermediate filament could interfere with the LAP2α effects on the E2F/Rb axis [84,85,86]. Supporting this hypothesis, it was recently shown that a specific knockout of *LMNA* in the pancreas induced a significant reduction of the Rb expression level and consequently an activation of E2F, evidenced by the increased expression of its target genes [87]. Therefore, future research could evaluate whether this molecular process is involved in the regulation of the Rb-E2F axis during adipocytic differentiation and if *LMNA* mutations would interfere with it.

Finally, in a recent study, we characterized the effect of two new mutations in *LMNA*—p.R582L and p.G631D—associated with severe metabolic phenotypes [88]. Significantly, using RNAseq of the subcutaneous adipose tissue from patients carrying these newly identified mutations, we highlighted that they were associated with the upregulation of several genes implicated in immunity, such as *TREM2*. The *TREM2* gene encodes a type I transmembrane protein that is a member of the immunoglobulin (Ig) receptor superfamily [89]. The TREM2 protein promotes adipocytic differentiation by upregulating adipocytic regulators in conjunction with inhibiting the Wnt/β-catenin signaling pathway. In addition, TREM2 activates adipocyte-derived MCP-1 and the infiltration of F4/80+CD11c+ macrophages into adipose tissue, which is important for dead adipocytes and cellular content cleaning [90,91]. Recently, a novel and conserved TREM2+ lipid-associated macrophage subset was identified [92]. The authors underlined TREM2 signaling as a major pathway by which macrophages responded to the loss of lipid homeostasis at the tissue level, indicating that TREM2 is a key sensor of metabolic imbalance across multiple tissues. As the TREM2 targets, Wnt/β-catenin and MCP-1, were suggested to be involved in the physiopathology of FPLD2 associated with *LMNA* mutations, this Ig receptor appears to be a great candidate for investigation [37,38,93].

In summary, the mTOR and Rb-E2F signaling pathways, as well as the transmembrane protein TREM2, appear to be interesting targets for unraveling the mechanisms involved in MD in the context of *LMNA* mutations. Consequently, it is important to understand how lamin A/C modulates the activation of the mTOR axis and controls the Rb and TREM2 expression in physiological and pathological contexts. One interesting question that remains open relates to how FPLD2-associated mutations can lead to an abnormal body fat distribution. This is discussed in the next section.

### 2.6. Can the Biology of Adipose Tissue Explain the Physiopathology of FPLD2?

A characteristic feature of FPLD2 is the abnormal body fat distribution, which gives patients a cushingoid appearance. For instance, a regional loss of subcutaneous adipose tissue from the limbs, buttocks and trunk is observed in FPLD2 patients from puberty and is followed by progressive fat accumulation in the face, neck and axillary regions. Additionally, fat accumulation in the visceral region is characteristic of FPLD2, suggesting an abnormal repartition of fat storage. This could be explained by the origin of adipose tissues. 

It is well-established that two types of adipose tissues can be distinguished: White adipose tissue and brown adipose tissue. Lineage-tracing studies have demonstrated that brown adipocytes originate from a subpopulation of dermomyotomes, which are also muscle cell progenitors [94,95,96,97], while white adipocytes’ origin appears to be more heterogeneous [98,99,100,101]. For example, lineage-tracing of SOX10 cells, which are cells localized in the neural crest, during mouse development revealed that such cells are the origin for a subset of adipocytic cells located in the head, but not in the inguinal and perigonadal regions [101]. 

A hypothesis for the abnormal fat distribution could be that *LMNA* mutations alter the expression of genes involved in the lineage of white adipocytes and result in visceral fat deposition and upper-body subcutaneous adipose tissue accumulation [102]. A complementary hypothesis is that *LMNA* mutations also impair the balance between white and brown adipose tissues. This is supported by the study of Pelligrini et al. showing an impairment of large lipid droplet formation, altered regulation of adipose tissue genes and the abnormal expression of the brown adipose tissue marker uncoupling protein 1 (UCP1) in differentiating white adipocyte precursors [103]. Conversely, in lipodystrophic brown adipocyte precursors induced to differentiate, the authors noticed the formation of enlarged lipid droplets typical of white adipocytes and the dysregulation of brown adipose tissue genes. In agreement with these results, Béréziat et al. observed that adipose tissue from an FPLD2 patient’s neck, which is an area usually exhibiting brown adipogenesis, displayed a white phenotype [104].

In summary, these studies suggest that FPLD2-associated *LMNA* mutations could interfere with the development of both types of adipose tissues. Therefore, further studies should be directed towards understanding the molecular mechanisms underlying the lineage of adipocytes and the function that lamin A/C may have in these processes. Another axis to explore includes the mechanobiological processes in which the lamin A/C protein is central and the processes that could be involved in the development of laminopathy phenotypes. Such processes will be described in the next section, with attention drawn to the impact on MDs and cardiovascular phenotypes.

## 3. A Link between MDs and Cardiovascular Diseases and the Alteration of the Mechanobiological Response of Stem and Vascular Cells

Lamin A/C participates in regulating the nuclear mechanical properties and responses to extracellular stimuli. Indeed, the nuclear lamina is a major contributor to nuclear stiffness, and its ability to endure local forces is mainly supported by lamin A/C, as low levels of A-type lamins increase the fragility and risk of deformation of the nucleus [2,105]. As laminopathic cells display nuclear rheological properties (rigidity and viscoelasticity) distinct from those of normal cells, aberrant nuclear mechanical properties are considered to be involved in the physiopathology of laminopathies [106]. Consequently, some studies have focused on the impact of FPLD2-associated *LMNA* mutations on the mechanical behaviors of the two main cell types, namely, adipocytes and vascular cells.

### 3.1. Changes of the MSCs’ Mechanoresponse Induced by FPLD2-Associated LMNA Mutations Inhibit Adipocytic Differentiation

In the last decade, an increasing number of publications have identified mechanosignaling as a major path to stem cell differentiation [107,108,109]. Among these studies, some were interested in understanding the mechanical processes associated with MSC differentiation into mature adipocytes. The role of the ECM in MSC differentiation was first highlighted by a study showing that tensile straining of a collagen I substrate stimulated osteogenesis and decreased the adipocytic differentiation of adhered MSCs [110]. This was associated with the mitogen-activated protein (MAP) kinase signaling pathway, since the inhibition of mitogen-activated protein kinase (MEK) decreased the osteogenic gene expression and matrix mineralization, while blocking the strain-induced downregulation of non-osteogenic lineage marker genes. These results were replicated by multiple works in vitro [111,112,113,114,115,116]. Other signaling axes were also involved in the inhibition of the adipocytic differentiation under mechanical stress, namely, the Wnt/β-catenin, transforming growth factor beta (TGF-β1)/Smad2 and yes-associated protein (YAP)/Tazzafin (TAZ) signaling pathways [111,112,115,116,117]. 

Additionally, ECM stiffening was linked to an enhancement of osteogenesis and an inhibition of the adipocytic differentiation of MSCs [111,116,118]. When MSCs were grown on a soft matrix, the percentage of cells that differentiated into adipocytic cells was higher than when grown on a stiff matrix. Remarkably, a knockdown of lamin A/C in MSCs greatly favored their differentiation into adipocytic cells on a soft matrix [41]. These observations are in line with the observation of a lamin A/C expression level decrease upon 3T3-L1 preadipocyte differentiation [93]. From this knowledge, it has been proposed that the lamin A/C meshwork in MSCs could respond to forces applied by 2D substrates and 3D microenvironments by changing its structure, expression level and localization. Such changes would in turn affect the MSC differentiation state [41,119,120,121,122]. How lamin A/C mutations influence MSC differentiation through changes in the lamina’s mechanical properties is currently not well-understood, but various sets of experiments have revealed some clues. 

An initial study suggested that the lamin A/C level regulates the tissue elasticity in mouse and human organs, through a mechanism that modulates gene expression and involves matrix-directed stem cell lineage determination [41]. In this line, several other studies linked *LMNA* mutations to the alteration of the ECM resulting from the expression and activation of diverse metalloproteases known to affect the ECM integrity [31,104,123,124,125,126]. 

The pioneer work of Worman’s group demonstrated that mutations responsible for FPLD2 and atypical lipodystrophies were associated with imbalances of TGF-β and ECM in adipose tissue [126]. Using mice specifically overexpressing the lamin A/C R482Q mutant in adipose tissue, the authors observed alterations of the ECM composition in this tissue similar to those reported from patients with FPLD2. Human and mouse subcutaneous adipose tissues expressing this mutant displayed increased fibrosis and a decreased mean adipocyte area. The expression levels of three other components of the ECM were also modified: (1) The expression of fibronectin, which binds collagen I and is involved in the maintenance of adipocyte shape, was increased, whereas the expression of both (2) elastin, a major component of elastic fibers, which provides strength and flexibility to connective tissue, and (3) decorin, which also binds to collagen I and participates in matrix assembly, was decreased. Comparable ECM aberrations were observed in cultured fibroblasts from patients with FPLD2 or other *LMNA* mutations associated with MDs. These anomalies were linked to the activation of TGF-β signaling, which is a driver of matrix deposition, and to an increased expression level and activity of metalloprotease 9, which is an endopeptidase that degrades ECM proteins. 

Additionally, stromal cells isolated from the adipose tissue of young, adult and old mice were used to test the hypothesis that mechanical loading modifies senescence-related changes in the self-renewal, osteogenic and adipocytic differentiation potentials [127]. To test the hypothesis, cells were subjected to 48 h of cyclic strain and reseeded and osteogenic differentiation was induced for various periods of time. Remarkably, such experiments highlighted that mechanical loading counteracted senescence-dependent changes, such as the decrease of cellular self-renewal. Mechanical loading also significantly reduced the number of oil droplets and the expression of adipocytic marker genes in cells from adult and old individuals. Hence, the study demonstrated that mechanical stretching, mimicking environmental stiffening, interfered with the decrease of stromal cell self-renewal and, at the same time, reduced adipocytic differentiation in cells from aging mice. As it was suggested that *LMNA* mutations induced the premature senescence of MSCs, reduced tissue stiffness and modified cell mechanobiological properties, this supports a functional link between *LMNA* mutations and MSC exhaustion and the fate of differentiation.

In summary, by altering the integrity of the ECM and by inducing premature senescence, *LMNA* mutations could affect the stem cell lineage determination and consequently interfere with the proper differentiation of adipocytic cells in FPLD2 associated with mutations of *LMNA*. As MDs are a common feature of laminopathies, it is important to increase our knowledge concerning the cell mechanobiological property changes linked to *LMNA* mutations and their effects on cell differentiation and lineage. The identification of new mechanosignaling pathways involved in the physiopathology of MD represents a major avenue for identifying new potential therapeutic targets for FLPD2 and other laminopathies. Vascular cells are also a cell type responding to mechanical stress from their environment and they exhibit an altered mechanobiological response when affected by *LMNA* mutations. This is the subject of the next section.

### 3.2. LMNA Mutations Associated with FPLD2 Physiopathology Interfere with the Precise Response of Vascular Cells to Shear Stress

Flowing blood generates a friction force called shear stress that has major effects on the vascular function. While high and laminar shear stress is considered to be physiological, low or oscillatory shear stress, mainly observed at bifurcations and bends of arteries, is associated with a mechanical configuration that promotes vascular dysfunction and atherosclerosis [128,129]. Endothelial cells are crucial sensors of shear stress; however, the mechanisms by which they decode complex shear stress environments to regulate physiological responses is currently only partially understood. Lamin A/C was described as being involved in these processes as a component of shear stress sensing, but also as a source of physiopathological responses when mutated.

The innovative studies by Jiang’s group revealed that low shear stress upregulated migration and proliferation, increased the production of platelet-derived growth factor (PDGF) and TGF-β1, enhanced the expression level of lysyl oxidase and phospho-ERK1/2 (extracellular signal-regulated kinases 1 and 2) and decreased the lamin A/C expression level in endothelial and vascular smooth muscle cells [130]. Additionally, the authors explored the effect of lamin A/C and emerin on the response of vascular smooth muscle cells to low shear stress [131]. They observed that low shear stress led to an increase in the cell population concomitant with a decrease in the amounts of lamin A/C and emerin. Using ChIP-on-ChIP experiments, the authors demonstrated that both lamin A/C and emerin modulated the activation of various transcription factors, including E2F1 and Sp1, involved in smooth muscle cell proliferation. These two studies were recently completed by work showing that the downregulation of lamin A/C was mediated by miR-124-3p, which bound to the 3’UTR of lamin A/C mRNA in vascular smooth muscle cells under pathological low shear stress and led to cell apoptosis [132]. In this model, lamin A/C repression affects the activity of p53, cAMP receptor protein responsive element binding protein 1 (CREB1), myc, a signal transducer and activator of transcription (STAT1/5/6) and Jun, which may in turn activate apoptosis. Later, a study confirmed that low shear stress led to a significant reduction in the lamin A/C expression level, which was associated with an increase in the proliferation and apoptosis of endothelial cells, depending on STAT1/3/5/6 activation [133]. In addition, although lamin A/C downregulation is not essential for glucocorticoid receptor nuclear translocation, it is important to properly regulate the glucocorticoid response element transcriptional activation in response to shear stress, which represses the inflammatory response of endothelial cells [134]. 

Different groups have investigated the impact of *LMNA* mutations on the mechanobiological properties of vascular cells. Notably, Olric’s group compared ascending aortas from WT and progeria mice after 30 min of high shear stress and showed that the expression levels of several mechanotransduction proteins in the aorta, including vimentin, transgelin and vinculin, decreased in mutant mice, while no effects were observed for WT mice [135]. 

Andrés’s group investigated the mechanisms controlling vessel stiffness in progeria mice with ubiquitous progerin expression and in mice expressing progerin specifically in endothelial cells and vascular smooth muscle cells, in order to determine the specific contributions of each cell type to vascular pathology [136,137]. Structural alterations in the aortas of progeroid mice were associated with a decreased smooth muscle tissue content, increased collagen deposition and decreased transverse waving of elastin layers in the media. Functional studies identified collagen as an underlying cause of aortic stiffening in progeroid mice. Consistently, an increased deposition of collagens III, IV, V and XII in the media of progeroid aortas was found. Therefore, the study suggested that progeroid arteries exhibited a higher stiffness and inward remodeling, mainly due to progerin-induced damage to vascular smooth muscle cells, which caused an increased deposition of medial collagen and a secondary alteration in the elastin structure. 

Complementarily, Foisner’s group investigated the contribution of the endothelium to cardiovascular alterations by generating a mouse model selectively expressing progerin in endothelial cells [138]. The authors described that endothelial cells expressing progerin displayed an impaired shear stress response through the alteration of nucleocytoskeletal coupling, the increase of F-actin/G-actin ratios and the deregulation of mechanoresponsive myocardin-related transcription factor-A (MRTFA). Thereby, progerin expression led to changes in mechanoresponsive components at the NE, mediating a profibrotic paracrine response of fibroblasts. 

Truskey’s group produced an impressive model for studying the impact of progerin on vascular cells’ physiopathological response to shear stress. The authors elaborated a functional 3D model of progeria that replicated an arteriole-scale tissue engineered blood vessel (TEBV) using smooth muscle and endothelial cells derived from a progeria patient fibroblast induced in pluripotent stem cells [139]. By exploiting this model, they showed that progeria-derived endothelial cells aligned with the flow, as non-mutated cells do, but exhibited reduced flow-responsive gene expressions [140]. Compared to TEBV engineered with healthy cells, progeria-TEBV exhibited reduced capacities, including vaso-activity and dilation capacities, and presented markers of cardiovascular disease. Finally, the progeria-derived endothelial cells produced vascular cell adhesion protein 1 (VCAM1) and E-selectin proteins, while healthy-derived cells did not. 

In summary, these studies altogether suggest that, in pathological conditions, the lamin A/C expression level is significantly reduced, which participates in the major reprogramming of gene expression through the activation of key transcription factors by endothelial and vascular smooth muscle cells. However, as the mechanisms underlying lamin A/C’s action on cell mechanobiological behavior under physiological conditions remain unclear, the specific effects of *LMNA* mutations driving FPLD2 physiopathology on the responses of vascular cells facing shear stress are poorly understood. Deciphering the impact of *LMNA* mutations on cell mechanics could be an excellent way to develop new therapeutic and diagnostic strategies. Therefore, in the last section, we will summarize several tools used to analyze cellular mechanical properties and introduce one of them as a potential diagnostic tool.

## 4. Microfluidic Tools to Investigate Alterations of the Cellular Mechanical Properties Induced by FPLD2-Associated *LMNA* Mutations

Mechanics clearly play a crucial role in numerous biological processes, among which are the nucleus mechanotransductional processes, leading to constantly deeper exchanges between the physics and biology communities. From a physics point of view, how a material responds to various mechanical stimuli is defined by a set of parameters broadly referred to as its ‘mechanical properties’. According to these parameters, cells are considered viscoelastic material, whose deformation under constraints simultaneously stores and dissipates mechanical energy. To understand the coupling between mechanical stress and biochemical signaling at play in cell responses to constraints, physicists have developed various experimental techniques, which are applied at the single cell and multicellular scales. 

Interpretation of the data requires the development of models, which, similarly, can span from molecular interactions to cell assemblies considered as a continuous medium. The underlying goal of current cell biomechanics research is to combine theoretical, experimental and computational approaches to construct a realistic description of cell mechanical behaviors that can be used to provide new perspectives on the role of mechanics in diseases. In particular, the understanding of the effects of *LMNA* mutations on cell mechanical properties is crucial for uncovering the mechanisms of the physiopathology of laminopathies and the associated MDs and cardiovascular diseases. In the long term, apparatuses used to analyze cell mechanical behaviors, once they are proven to be able to highlight the differences between healthy and lamina-deficient cells, could be used in research experiments, but also as diagnostic tools. Indeed, beside the typical FPLD2, *LMNA* mutations have also been involved in a few cases of severe metabolic syndrome. These attenuated forms of laminopathies, given the high proportion of patients with metabolic syndrome or metabolic abnormalities with an early onset in the general population, could be under-diagnosed as gene testing is not systematically performed [74].

### 4.1. Powerful Tools for Assessing Cell Mechanicanical Properties Based on Their Capacity to Deform Themselves or Their Environment

There are now a myriad of experimental techniques and theoretical models that have been adapted from soft matter physics and mechanical engineering to characterize cell mechanical properties. The mechanical measurement techniques can be separated into two main categories: (1) Those assessing the cell’s ability to generate forces, and (2) those used to apply forces on a part of, or on the whole, cell [141]. 

**Figure 2:**
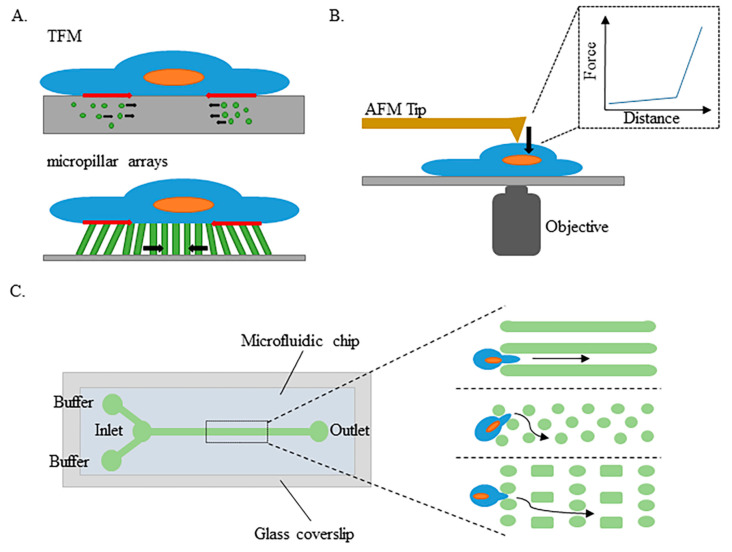
Schematics of four tools used to explore cellular mechanics. (**A**) Traction force microscopy (TFM) and micropillar arrays are techniques focusing on the ability of cells to apply forces and deform their environment. By following the displacement of the probes (motion of fluorescent beads (in green) in the soft substrate or deflection of fluorescent pillars), experimenters can determine the forces applied by cells to deform their substrate. (**B**) Atomic force microscopy (AFM) allows the viscoelastic properties of cells to be determined by measuring the relationship between the AFM tip displacement and the force applied to deform the cell (right part of the graphics). (**C**) Different designs of microfluidic chips allowing studies of dynamic cell deformation through small passages can be used to study the viscoelastic response of cells.

The first category of techniques focuses on measuring the forces a cell can exert on its environment (Figure 2A): This is the case for traction force microscopy (TFM), where forces are inferred from the motion of probes embedded in the soft 2D substrate or 3D surrounding matrix [142], and of micropillar arrays, where the deflection of elastic pillars on the substrate that the cell adheres to indicates the amplitude of the cell traction force [143,144]. Such experiments, commonly used to study cell adhesion and migration, allow the establishment of the local force field and deciphering of the spatiotemporal coordination of the cell. However, the accessibility to the mechanotransduction processes and the nucleus behavior using this type of technique is very low. 

The second category of techniques consists of applying a controlled force on the cell or a part of the cell and measuring the resulting deformation (see the example of the atomic force microscopy (AFM) technique in Figure 2B), either locally via attached beads (magnetic bead cytometry and optical tweezers) or at the scale of the whole cell (micropipette, cell stretchers, microfluidics and AFM). The correlation between stress and strain depends on the mechanical properties of the cell. The local techniques, involving magnetic and optical tweezers, allow for local and global deformation, with magnetic tweezers reaching higher forces (in the tens of nN range) than optical tweezers (less than 100 pN), and lead to similar viscoelastic moduli values [145,146,147,148]. The other setups allow deformation of the entire cell (Figure 2B). From the different deformation regimes observed, one can deduce the cell rheological properties using one of the numerous mechanical models. Micromanipulation with a micropipette is a traditional technique that has been used for decades to aspirate single cells, mostly blood cells [149,150], as well as cell aggregates [151]. AFM was used to probe the viscoelastic response of cells and reveal the specific cytoskeleton dynamics [152]. Recently, a cell stretcher was used to show that the actin cap protects the nucleus structural integrity from substrate-induced stress [153]. Another study used the cell stretcher to elegantly describe an unconventional role of chromatin in altering its own mechanical state to maintain genome integrity in response to deformation [154]. As the models used to interpret data were all based on assumptions on the contributions of the various components of the cell, the cell properties varied substantially between the studies, suggesting differences in how the results of different methods were obtained or analyzed [155]. To establish the validity of the measured changes, it is thus crucial to compare data obtained with the same type of experiment.

The main drawbacks of most of these techniques are the low throughput, such as that of a micropipette and AFM (which consist of single-object experiments, thus being time consuming and limiting the statistics), and the fact that they require extensive training of the manipulator, such as the micropipette, AFM, or optical tweezers. The recent development of microfluidics tools, in the past two decades, has renewed the field of cell biomechanics. Indeed, this rheology flow method, which consists of studying the flowing motion and deformation of objects under a controlled flow, in networks of channels with dimensions ranging from tens to hundreds of micrometers, uses small quantities of materials (in the range of microliters), and is compatible with live observation under a microscope. This method allows multiple object handling (with several channels in parallel), though it is still not a high-throughput method (Figure 2C). Regardless of this, it is very informative in terms of an object’s dynamic behavior and allows both qualitative and quantitative analysis [156]. This technique is, therefore, being used increasingly frequently to investigate the rheology of various cell types, from blood cells [157,158,159] to dendritic cells [160]. 

From the studies described in Section 3, it is clear that microfluidics would be a powerful tool for measuring the viscoelastic properties of cells and nuclei from laminopathic patients. Both cell and nucleus contours can be tracked over time as the object passes through a micrometer-sized channel (Figure 2C), and the flow can be tuned to determine a critical pressure to force the object into the channel. Alterations of the mechanical behavior induced by *LMNA* mutations are expected to be detected when cells have to deform in order to pass through narrow channels. As previously noted, a physical model of the cell and its nucleus rheology must be developed to interpret the cell passage and deduce the mechanical properties. With the channel design being versatile (Figure 2C), one can constrain the cells once with one constriction [160], or several times with successive constrictions [159,161]. In addition to following a single deformation and relaxation dynamics, such configurations allow for assessing whether the cell has an adaptive ability and modifies its behavior as it progresses further in the channel network, as was suggested for monocytes smoothly transiting through tens of successive capillaries in the pulmonary bed [159].

### 4.2. Towards an Easy Diagnosis for Metabolic Disorders Associated with LMNA Mutations

In the context of diagnosis, one of the primary concerns is obtaining a solid quantification with a high reproducibility and a cheap cost, meaning that a great number of cells have to be analyzed and experimental elaboration, as well as analysis, must be easily automatized. The majority of the techniques described above are adapted for single cell-type experiments with low statistics, and some are expensive, either in terms of equipment and/or in terms of manpower. For example, optical tweezer micromanipulation and AFM deformation currently cannot be adapted for low-cost measurements. 

The cheapest method is currently microfluidics. Despite the variety of possibilities, the most common materials used for the fabrication of microfluidic devices are silicon wafers, glass and polymers. In general, microchannel fabrication techniques are specific to the materials employed; however, the most common method is based on photolithography, which can be highlighted as the most popular technique due to rapid prototyping and replica molding. Based on this method, soft lithography is widely employed to fabricate microchannels made of soft polymers (most often polydimethylsiloxane (PDMS)). Microfluidic tools are highly modular as the channel design is versatile in size and geometry: For instance, channels can be straight, bent, spiraled, alternately bent, expanded and/or reduced (Figure 2C). 

Using microfluidics methods, it should be possible to determine parameters that specifically distinguish the effects of each known mutation of *LMNA* on the cell and nucleus susceptibility to deform, and establish a phase diagram correlating the cell mechanical behavior to the disease. Studies have been conducted on cells carrying *LMNA* mutations, which displayed an increased viscosity and stiffness measured by AFM [162,163]. Another study described that the branched actin network, connected to the nucleus through the LINC complex, facilitates cell passage through microfluidic constrictions [160]. This involves the weakening of the lamin A/C network, which favors nuclear deformation. As *LMNA* mutations have heterogeneous effects from the cellular to the clinical level, developing quantitative data based on the deformation ability of cells and the nucleus might be helpful for classifying the severity, as well as diagnosing the clinical effects of such mutations. One of the most common hypotheses is that the severity of biomechanical defects due to *LMNA* mutations correlates with the severity of the clinical phenotype. 

An aim would also be to implement the microfluidics experiment as a diagnosis tool to assess the nucleus and cell deformability from patients with an unknown condition. In combination with genome sequencing, this might be helpful for rapidly identifying new mutations related to MD and/or cardiovascular diseases known to be common features of laminopathies and the ones characterizing FPLD2. Another use could be testing new treatments for FPLD2 or MD and cardiovascular diseases associated with laminopathies, which are still very rare as there are few approaches for assessing their efficiency.

## 5. Conclusions

**Figure 3:**
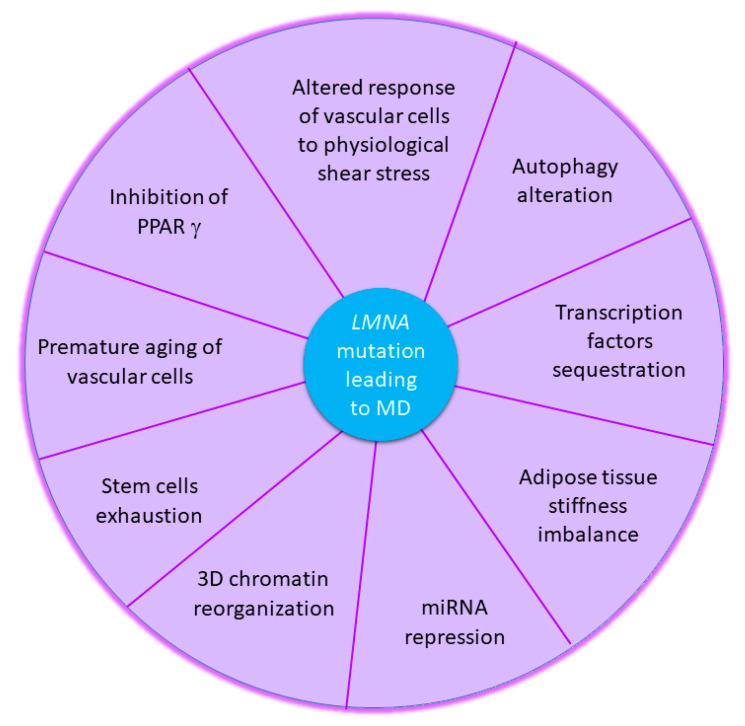
Cellular and molecular hallmarks of metabolic disorders associated with *LMNA* mutations.

In conclusion, MDs associated with *LMNA* mutations represent complex conditions from the clinical to the cellular and molecular levels. The hallmarks of MD that we described in this review are clearly linked to lamin A/C function alterations (Figure 3). Therefore, in addition to providing advances in understanding the molecular processes leading to lipodystrophy, these various works have also informed us about the lamin A/C physiological functions. Among the lamin A/C properties highlighted in this review, one of the most interesting, and which deserves further investigation, is the capacity to act as a sensor and signaler of mechanical stress. Studying the effects of *LMNA* mutations on the cell mechanical properties would allow the scientific community to improve the knowledge regarding how a single mutation can affect one or several important tissues. Additionally, exploring these property alterations as a hallmark of MD could represent a major advance in the development of diagnostic tools, as well as new therapeutic avenues.
